# Duration of storage does not influence pregnancy outcome in cryopreserved human embryos

**Published:** 2013-10

**Authors:** Nastaran Aflatoonian, Soheila Pourmasumi, Abbas Aflatoonian, Maryam Eftekhar

**Affiliations:** 1 *Madar Hospital, Yazd, Iran.*; 2 *Research and Clinical Center for Infertility, Shahid Sadughi University of Medical Sciences, Yazd, Iran.*; 3 *Department of Obstetrics and Gynecology, Research and Clinical Center for Infertility, Shahid Sadughi University of Medical Sciences, Yazd, Iran.*

**Keywords:** *Cryopreservation*, *Assistedreproductivetechnology(ART)*, *Pregnancyoutcome*

## Abstract

**Background:** Cryopreservation of embryos has been an usual component of clinic in assisted reproductive technology (ART) programs. Recently the dramatic increase in cryobiology activity in the clinical centers has enhanced methods of freezing and improved vitrification protocols are being developed.

**Objective:** The aim of our study was to assess the effect of storage duration of frozen embryo on ART outcome.

**Materials and Methods:** In this retrospective study the data of 651 frozen-thawed embryo transfer cycles were assessed over a 36-months period. Our patients were categorized according to storage time of freeze. Group I: less than 90 days, Group II: between 90-365 days. Group III: between 365-730 days. Group IV: between 730-1095 days. Group V: more than 1095 days. Clinical pregnancy and implantation rate were defined and statistical analysis was performed using Student t-test and Chi-square.

**Results:** According to our finding patient’s mean age was 31.05±5.231 years (range, 18-53 years), and 1204 embryos were transferred .The mean storage duration was 296.72±301.82 days. The mean number of embryo transferred per cycle was similar between groups (p=0.224). According to our analysis clinical pregnancy rate per embryo transfer cycle was similar between groups (p=0.563).

**Conclusion:** Our results showed that duration of storage had no negative effects on implantation of cryopreserved embryos. In our literature review we found a little article In this context. However our study showed duration of freezing don’t have any negative effects on implantation and pregnancy outcome, but more studies are needed to evaluate long term effects of storage duration on babies were born by cryopreserved embryos.

## Introduction

Cryopreservation of embryos has been an usual component of clinic in assisted reproductive technology (ART) programs (1). For most of the time in ART cycles where ovarian stimulation in combination with ART, results in a large number of embryos, it offers the opportunity to reduce the number of transferred embryo per cycles, and thus limits the risk of multiple pregnancy, in patients that are in risk of ovarian hyper stimulation syndrome (OHSS). Freeze of all embryos will decrease the risk of OHSS, also when endometrium is not prepared for embryo transfer it is suggested to cryopreserve all of the embryos and then transfer them in an ideal condition (2-5). Embryo freezing can lead to loss of some embryos but is not reported to have any delayed effect. However, there is some concern about this viewpoint (6). 

Several studies concluded that embryo freezing can effect on early stage of embryo development and influence on nuclear DNA, mitochondrial genome and early steps of cell division such as transcription , translation and imprinting (7). During cryopreservation process inactivation of enzyme, ionic imbalance and generation of free radicals could damage these critical processes (6). With respect to destructive effects, some studies have recommended the probability of increasing rates of post-implantation losses as showed in humans and mice (8, 9). 

The number of cryopreserved embryos in storage has increased, as well as the amount of time in storage (10). Today the intense rise in cryobiology activity in the clinical centers has enhanced methods of freezing and improved vitrification protocols are being developed (7, 11).

There are few studies on the effects of the storage duration on frozen embryos. The first study by Testart *et al* showed an increase in the rate of embryonic cell loss after a few months of storage (12). However, Cohen *et al *reported that the increased duration does not have an effect on potential development of embryos (13). The aim of our study was to assess the effect of storage duration of frozen embryo on ART outcome. 

## Materials and methods

This cross sectional study was conducted at Madar Hospital, over a 36-months period between January 2009 and January 2012. 651 couples were participated in the study .All women had previously undergone in vitro fertilization (IVF) or intracytoplasmic sperm injection (ICSI) with embryo cryopreservation. Women with age >39 years, body mass index (BMI) >30 kg/m^2^, history of endocrine disease were excluded from the study. This study was approved by ethics committee of Research and Clinical Center for Infertility, Shahid Sadoughi University of Medical Sciences.

**Embryo cryopreservation and transfer**

Morphological evaluation of all embryos is done on the second day after ovarian puncture; blastomeres are counted and cytoplasmic fragmentation were assess. In our clinic more than three embryos is not transferred in fresh cycles and all the surplus embryos with <30% fragmentation are cryopreserved by vitrification method.

After a two-steps loading, with equilibration solution containing dimethyl sulfoxide and ethylene glycol and vitrification solution containing dimethyl sulfoxide, ethylene glycol and 0.5 mol/L sucrose, embryos are loaded by a thin glass capillary tube on the cryotop. After loading, nearly the whole solution is eliminated and only a fine layer covered the embryos, and the samples were immediately submerged into liquid nitrogen. Then the film part of cryotop is covered by a plastic cap, and the sample is stored under liquid nitrogen.

Thawing is done at least 2 months after cryopreservation. Straws are exposed to warm water bath in 37^o^C for 30 s; cryoprotectants are eliminated step by step using embryo-thawing media (Vitrolife, Sweden). Embryos are transferred to culture media before being evaluated for the number of survived blastomeres. Frozen-thawed embryos are considered morphologically survived by 50% or more intact blastomeres and no sign of injury to zona pellucida, embryos are cultured in media for only 1 day.

Endometrial preparation is done by Estradiol valerate (Estradiol Valerate, Aburaihan CO, Tehran, Iran) which was taken orally at the dose of 6 mg per day from the second day of menstrual cycle. Ultrasound examination is started from day 13 of menstrual cycle. It is used to assess endometrial thickness which is measured at the greatest diameter in the fundal region. When the endometrial thickness reaches more than 8 mm, 100 mg progesterone in oil (Progesterone, Aburaihan, CO, Tehran, Iran) is injected daily or cyclogest vaginal pessaries (Activis, Barnstaple, UK) 400 mg twice daily. Estradiol and progesterone administration are continued until the documentation of fetal heart activity by ultrasound. Thawing of the embryos is performed 2 days after the beginning of progesterone injection. Embryos are transferred 1 day and 3 days after thawing, respectively. The transfer was performed by a Labotect catheter (Labotect, Gottingen, Germany).

Our patients were categorized according to storage time of freezing. Group I: less than 90 days, Group II: between 90-365 days. Group III: between 365-730 days. Group IV: between 730-1095 days. Group V: more than 1095 days. Clinical pregnancy was defined while fetal heart activity was checked by trans-vaginal ultrasonography 5 weeks after positive beta hCG. Abortion was defined as loss of pregnancy before 20 wk of gestation. Ongoing pregnancy was defined as pregnancy was proceeded beyond the 12^th^ gestational week and implantation was defined by the number of gestational sacs per 100 transferred embryos.

**Statistical analysis**

Statistical analysis was carried out using the statistical package for the social science version 15.5 for windows (SPSS Inc., Chicago. IL, USA). Between-group differences of normally distributed continuous variables were assessed by Student’s t test. Significant differences were evaluated by the Chi-square test to compare the non-continuous variables. The data were expressed as mean±SD. P-value<0.05 was considered statistically significant.

## Results

A total of 651 patients who underwent frozen-thawed embryo transfer cycles were included in this study. The patient’s mean age was 31.05±5.231 years (18-53 years), and 1204 embryos were transferred. 

The mean storage duration was 296.72±301.82 days. The mean number of embryo transferred per cycle was similar between groups (p=0.224). According to our analysis clinical pregnancy rate per embryo transfer cycle was similar between groups (p=0.563). There was no statistically significant difference between implantation rate (p=0.988) (Table I).

**Table I:** ART outcome between different groups

**Variable**	**group I** **(n=189)**	**group II** **(n=312)**	**group III** **(n=92)**	**group IV** **(n=33)**	**Group V** **(n=25)**	**p-value**
No. of transferred embryo	1.88±0.586	1.80±0.637	1.82±0.566	2.03±.585	1.92±0.49	0.224
Implantation rate [%]	20.48±0.44	19.55±0.39	19.20 ±0.34	16.67±0 .34	18.00 ±0.24	0.988
Clinical pregnancy rate [(n) %]	(47) 24.9%	(70) 22.6%	(25) 27.2%	(7) 21.2%	(9) 36.0%	0.563

Parameters expressed as mean±SD or percentage as appropriate.

Chi-square and Student’s *t* test were used.

## Discussion

The aim of our study was to evaluate the effect of storage duration of cryopreserved embryo on ART outcome. Our result showed that duration of storage had no negative effects on implantation of cryopreserved embryos. In our literature review we found a little articles. In this context similar to our study Ashrafi *et al* in their study evaluated the factors affecting the outcome of frozen thawed embryo transfer cycle. They showed that there was no statistically differences in pregnancy rate when storage time of cryopreservation was less or more than 180 days (14). 

Wilson *et al* reported in their research about effect of the length of time that donated embryos are frozen on pregnancy outcome Longer time of freezing did not adversely affect subsequent pregnancy rates following frozen embryo transfer (15). The longest time of freezing of embryo lead to live birth reported by Dowling *et al* in 2010 from a frozen-thawed pronuclear stage embryo almost 20 years after its cryopreservation (10). Testart *et al* in their study showed increase rates of human embryonic cell loss with storage of several months (12). In contrast Cohen *et al* demonstrated no deleterious effects from storage (13). Surprisingly few clinical data are available to address this clinically relevant question. 

Some studies have evaluated the impact of cryopreservation on the implantation potential regardless of the storage duration of frozen embryos and concluded that freeze of embryo did not affect implantation rate. The duration of storage embryos could remind subject to legislation which be unlike in different countries (16). In Iran legislation regarding the duration of freeze does not exist. Therefore in this concern decisions regarding the duration of conservation excess embryos is made by the ART clinics. However There are some concerns in this context, three facts suggest that embryo freezing might be a matter of worry: an increase of free radicals due to cryopreservation process (17). Formaldehyde is a cytotoxic and mutagenic chemical substance, is used in cryoprotectant solutions and the toxicity of some cryoprotectant, such as dimethylsulfoxyde (DMSO), on genome.

The current idea that embryo freezing has no late effect relies on the results of previous studies, and on common experience in humans and animal. Recently, however, injury to the genetic material from cryopreservation has been demonstrated. Beside, in domestic species as in humans, researches have mainly focused on obvious defects at birth or in children. 

However our study showed duration of freezing doesn’t have any negative effects on implantation and pregnancy outcome, but more studies are needed to evaluate long term effects of storage duration on babies were born by cryopreserved embryos.
